# The Relation Between Free Testosterone and Components of Metabolic Syndrome in Women With Polycystic Ovary Syndrome 

**Published:** 2018-03

**Authors:** Zahra Abbasi-Ranjbar, Seyedeh Hajar Sharami, Soudabeh Kazemi, Daniyal Sayyad-Abdi, Seyedeh Fatemeh Dalil Heirati

**Affiliations:** 1 Department of Endocrinology and Metabolism, Reproductive Health Research Center, Guilan University of Medical Sciences, Rasht, Iran; 2 Reproductive Health Research Center, Guilan University of Medical Sciences, Rasht, Iran; 3 Guilan University of Medical Sciences, Rasht, Iran

**Keywords:** Testosterone, Polycystic Ovary Syndrome, Metabolic Syndrome, Hyperandrogenism

## Abstract

**Objective:** To assess the relationship between free testosterone level and components of MS in women with PCOS.

**Materials and methods:** This is a cross-sectional study which was conducted on 215 women with PCOS. PCOS was diagnosed based on the Rotterdam criteria. Patients were divided into two subgroups of patients with and without MS based on ATP III criteria. In each subgroup, the association between individual components of MS with free testosterone was measured. Data were analyzed using SPSS software.

**Results:** The prevalence of MS was 28.8% (n = 62). The mean level of free testosterone in patients with blood pressure ≥ 130/85 was significantly higher than those with blood pressure < 130/85 mm/hg. (p = 0.029) Also, in patients with diastolic blood pressure ≥ 85, the level of free testosterone was significantly higher than patients with diastolic blood pressure < 85. (p = 0.026). Results showed significant positive correlation between the level of free testosterone and cholesterol (p = 0.024). But no significant correlation was noted between levels of free testosterone and other variables.

**Conclusion:** Regarding the relationship between blood pressure and high levels of free testosterone, it seems that regular blood pressure screening has a higher priority of concern comparing other complications for preventing cardiovascular adverse effects in women with PCOS and hyperandrogenism.

## Introduction

Polycystic ovarysyndrome (PCOS) manifests as anovulation, oligomenorrhea and hyperandrogenism with the prevalence of 5-10% in women of childbearing age (1). Women with PCOS are at high risk of insulin resistance and hyperandrogenism, which can affect the irmiddle-age quality of life and cause long-term complications.

As, long-term consequences of PCOS including metabolic disorders and cardiovascular diseases increasingly can be seen, in recent decades, investigators noticed the metabolic aspects of PCOS (2-4). Metabolic syndrome (MS) is one of the most common disorders in these patients which can be characterized by central obesity, insulin resistance and impaired glucose metabolism (5, 6).

Regarding the relative similar pathogenesis of both diseases, their co- occurrence can be expected and early identification can help early detection and reduce their complications.

Cumulative evidences have shown that high androgen level was a constant risk factor in the pathogenesis of PCOS. High levels of testosteroneassociated with obesity, especially abdominal fat, insulin resistance and higher incidence of glucose intolerance (7, 8). Also, it has been reported that hyperandrogenism and insulin resistance increased testosterone production in the ovaries of women with PCOS (9). In the last clinical guideline of endocrinology, it has been suggested to use high levels of total and free testosterone for the diagnosis of PCOS (10).While previous investigations supported measuring androstenedione and testosterone as useful predictors of metabolic risk in patients with PCOS, in patients with laboratory hyperandrogenism and obesity, lower levels of and rostenedione and the increased ratio of testosterone to androstenedione were significantly shown (11-13).

Furthermore, Lerchbuam et al mentioned that PCOS women with higher levels of free testosterone and androstenedione did not have normal metabolic characteristics and also higher androstenedione to testosterone ratio accompanied with better metabolic profile (14).

Previous investigation demonstrated 5 fold increased risk of MS in patients at the highest quartile of free testosterone level but not for total testosterone. They recommended further studies to investigate the role of hyperinsulinemia as an interface for hyperandrogenism and MS (15).

Up to now, different results have been claimed and there is no definite androgen level in the diagnosis of MS. Therefore in this study with assessing the role of free testosterone in the metabolic profile of PCOS and normal women, we aimed to evaluate the androgens levels for predicting future MS and realizing the accuracy of preferred androgens.

## Materials and methods

This is a cross-sectional study which was conducted on 215 women with PCOS aged 15-35 years-old referred to gynecology and endocrinology clinics in Rasht during 2010-2012. The ethical approval was obtained from the Ethics Committee of Guilan University of Medical Sciences with number 189007505.

PCOS was diagnosed based on the Rotterdam criteria 2003 by the existence of 2 out of 3 following criteria: 1. Oligoovulation (menstrual cycles longer than 35 days, or less than 9 cycles per year), 2. Clinical hyperandrogenism (acne, Hirsutismor Ferriman-Gallwey Score ≥ 9) or laboratory hyperandrogenism (including total testosterone > 2.6 nmol/L, free testosterone ≥ 0.6pg/ ml), and 3.Morphological features of PCOS in ovarian ultrasound (≥ 12 antral follicles in each ovary measuring 2-9 ml, or ovarian volume greater than 10 cm^2^) (16).

After the necessary explanations, informed consents were obtained. Age, weight and height were measured and recorded for all patients. BMI was calculated by dividing weight by squared height. Waist circumference was measured by a tape meter from the top surface of iliac crest and just below the umbilicus. Hip circumference was measured in the horizontal plane and it was measured at the widest part. Systolic and diastolic blood pressure were measured in the sitting position after 10 minutes rest.

Patients with PCOS were divided into two subgroups of patients with and without MS based on ATP III criteria. Criterion of having the MS (16) was the occurrence of at least 3 risk factor out of 5:

Waist circumference ≥88 cm, triglycerides ≥ 150 mg/dl, HDL < 50 mg / dl, diastolic blood pressure ≥ 85 mm Hg and systolic blood pressure ≥ 130 mm Hg, and fasting glucose ≥100 or taking diabetes medication.

Women with hypothyroidism (TSH > 5), hyperprolactinemia ( serum prolactin ≥ 100ng/ml), congenital adrenal hyperplasia, androgen producing tumor, Cushing's syndrome, diabetes mellitus, and kidney disease were diagnosed by physical examination and assessment of TSH, PRL, 17α-OHP, TG, Cholesterol, HDL, LDL and FBS were excluded.

Finally, in each of these subgroups, the association between individual components of MS with free testosterone was measured.

***Statistical analyses:*** Data were analyzed using SPSS software version 21. Using independent t-test, mean testosterone levels between the patients with and without MS were analyzed. 

The difference between mean levels of testosterone among different components of the MS including waist circumference, blood pressure, fasting blood sugar, triglyceride levels and HDL levels were analyzed using independent t-test. 

**Table 1:** Comparing the demographic and pregnancy variables in PCOS women with or without MS

**Variables**			**Participants** **N = 205**	**With MS** **(n = 62)**	**Without MS** **(n = 153)**	**p value**
Age			25.63 ± 5.17	26.81 ± 6.07	25.15 ± 4.69	0.057
Place of inhabitant		Rural	34(16.2)	9(14.5)	25(16.9)	0.838
		Urban	176(83.8)	53(85.5)	123(83.1)	
Occupation		Employee	32(20.3)	11(24.4)	21(18.6)	0.511
		Housewife	126(79.7)	34(75.6)	92(81.4)	
Educational level		< Diploma	42(26.4)	12(27.3)	30(26.1)	0.950
		Diploma	56(35.2)	16(36.4)	40(34.8)	
		Academic	61(38.4)	16(36.4)	45(39.1)	
The history of infertility			95(54.9)	30(58.8)	65(68.4)	0.615
Duration of infertility (months)			36.50 ± 41.26	30.39 ± 24.04)	39.42 ± 47.21	0.705
History of abortion			13(10.6)	5(13.5)	8(9)	0.523
Family history of diabetes			83(39.3)	31(50.8)	52(34.7)	0.043
Parity	Nulipara		96(78)	24(75)	59(77.6)	0.760
	Multipara		27(22)	8(25)	17(22.4)	
Oligo-menorrhea			183(87.6)	52(89.7)	131(86.8)	0.647
Amenorrhea			20(9.3)	6(10.3)	14(9.3)	0.797

Using the Pearson correlation coefficient, correlation between testosterone levels and anthropometric and laboratory parameters were evaluated. P.value < 0.05 indicated statistical significance and 95% confidence interval was noted.

## Results

In this study, 215 patients with PCOS were assessed. The prevalence of MS was 28.8 percent (n = 62). The mean age of participants was25.63 ± 5.17. Most of the participants lived in urban area, were housewives and had academic education. There was no significant difference between groups regarding all variables except the family history of diabetes (p = 0.043) (Table 1). 

In reviewing the components of MS in all participants (215 patients), results showed that 72.6 % (156) had waist circumference ≥ 88 cm, 9.3% (20) had blood pressure≥130/85 mm Hg, 6% (13) had fasting blood sugar ≥110 mg per deciliter, 47 % (101 cases)had triglycerides ≥ 150 mg per deciliter, and 86 percent (185 cases) had HDL < 50 mg per deciliter. Table 2 compared the components of MS in patients with and without MS.

The mean testosterone level in patients with and without MS was not significant, however comparing with its components; mean free testosterone was significantly related with diastolic blood pressure and hypertension. Whereas in patients with diastolic blood pressure ≥ 85, the level of testosterone was significantly higher than patients with diastolic blood pressure < 85 (p = 0.026). 

Also, the mean level of testosterone in patients with blood pressure ≥ 130/85 was significantly higher than those with blood pressure < 130/85 mmhg. (p = 0.029). There was no significant difference between other components of MS and free testosterone level (Table 3).

Results showed significant positive correlation between the level of free testosterone and cholesterol (p = 0.024). But no significant correlation was noted between levels of free testosterone and other variables (Table 4).

**Table 2:** The frequency of MS components in patients with and without MS

**MS components**	**With MS** **(n = 62)**	**Without MS** **(n = 153)**
Waist circumference ≥ 88 cm	59(95.2)	97(63.4)
HTN (diastolic blood pressure ≥ 85 mm Hg or systolic blood pressure ≥ 130 mm Hg)	17(27.4)	3(2.0)
Fasting blood glucose ≥ 100	12(19.4)	1(0.7)
Triglycerides ≥ 150 mg / dl	55(88.7)	46(30.1)
HDL < 50 mg / dl	61(98.4)	124(81.0)

**Table 3:** Comparing mean free testosterone in patients with and without MS and its components

**Variable**		**Number**	**Free Testosterone level**	**p value**
Metabolic syndome	Yes	62	1.61 ± 1.14	0.805
	No	153	1.65 ± 1.09	
Waist circumference(cm)	≥ 88	156	1.68 ± 1.18	0.340
	< 88	59	1.54 ± 0.87	
Systolic blood pressure(mm Hg)	≤ 130	18	2.13 ± 1.37	0.510
	> 130	197	1.60 ± 1.06	
Diastolic blood pressure(mm Hg)	≤ 85	12	2.33 ± 1.39	0.026
	> 85	203	1.60 ± 1.07	
Blood pressure(mm Hg)	≤ 130/85	20	2.16 ± 1.35	0.029
	> 130/85	195	1.60 ± 1.07	
FBS(mg/dl)	≤ 110	13	1.17 ± 0.60	0.110
	> 110	202	1.67 ± 1.12	
TG(mg/dl)	≤ 150	101	1.65 ± 1.30	0.970
	> 150	114	1.64 ± 0.90	
HDL (mg/dl)	≤ 50	30	1.62 ± 1.07	0.615
	> 50	185	1.65 ± 1.11	

**Table 4:** the correlation between testosterone and anthropometric and laboratory indices

**Variables**	**Correlation ** **coefficient**	**p ** **value**
Waist circumference	-0.005	0.945
Body Mass Index	-0.086	0.209
Systolic blood pressure (mm Hg)	0.100	0.144
Diastolic blood pressure (mm Hg)	0.123	0.071
TG (mg/dl)	0.041	0.553
Cholesterol (mg/dl)	0.155	0.024
FBS (mg/dl)	0.028	0.680
LDL (mg/dl)	0.058	0.396
HDL (mg/dl)	0.018	0.794

## Discussion

One of the most common disorders in women with PCOS is MS, which increases the risk of cardiovascular disease and diabetes. PCOS women consistently had higher risk of developing MS compared to others. The prevalence of MS of Thai women was 18% and Apridonize et al mentioned 43%as the prevalence of MS in women with PCOS which was about twice higher than the 24% in NHANES population (17, 18).

Also, Mandrella et al and Rahmanpour et al mentioned 37.5% and 33.3% as the prevalence of MS, respectively (19, 20). It seems that the higher reported prevalence of MS might be noted regarding the diverse diagnostic and sampling methods

Also, PCOS significantly indicated as a significant health problem due to the higher prevalence of MS in women with PCOS, the overlap between metabolic and anthropometric disorders, and the association between MS and subsequent long-term increased risk of type 2 diabetes and cardiovascular disease.

The results of this study showed that comparing demographic variables between PCOS women with and without MS, 50.8% of patients with MS had a family history of diabetes. Also, 19.4% of women with PCOS had fasting sugar ≥ 100 which was higher than women without MS. However, no significant difference was noted between free testosterone and blood sugar which would be as a result of the exclusion criteria and the mentioned 19.4% belonged to Glucose intolerance. 

Studies showed that women with diabetes had higher level of free testosterone comparing women without diabetes. Therefore, reduction in the sex hormone binding globulin (SHBG) and high levels of free testosterone are accepted for the occurrence of diabetes type 2 and highlights the relationship between androgens and insulin sensitivity.

Reports revealed that free testosterone even in non-diabetic patients had a significant relation with hyperinsulinemia and hyperglycemia which are the components of MS and supported the androgen roles in the regulation of glucose homeostasis. There are several mechanisms that may link androgens and insulin resistance. Abdominal obesity by conversion of and rostenedione to testosterone by 17 beta-hydroxy steroid oxidoreductasecan itself cause hyperandrogenism, decrease in sex hormones binding globulin and increase the amount of free testosterone (8).

Previous Studies confirmed the relationship between MS and insulin resistance and hyperandrogenism. They mentioned high levels of androgens as a marker to identify MS (12). In a study by Kim et al, results showed 4-fold increased risk of MS in non-obese patients with high level of free testosterone and SHBG. But this association was not observed in obese patients (21).

Although in this study, no statistically significant difference was noted between the mean testosterone levels in patients with and without MS, in a review article by Muraleedharan et al inconsistent results were noted (22). They reported that low level of testosterone was significantly related with obesity, insulin resistance and inappropriate lipid profiles in men. Also in men with MS and type II diabetes, higher prevalence of hypogonadism was reported. As they reported MS and low level of testosterone as risk factors of mortality subsequent to cardiovascular diseases, testosterone substitution was recommended. 

Forrestar et al also demonstrated that MS in women with PCOS was not affected by hyperandrogenism. Because according to the diagnostic criteria for NIH which compared four high testosterone, moderate testosterone, high testosterone in obese and normal testosterone in thin participants, t-test noted no significant difference in glucose, lipid profile, LH and FSH, prolactin, and blood pressure (23).

Moulana et al observed that MS was associated with the occurrence of insulin resistance, dyslipidemia, hypercholesterolemia and diabetes type 2. Also, obesity and MS in men and women were associated with an increased risk of cardiovascular disease and hypertension. In men, obesity and MS associated with reduced level of testosterone and in obese women with MS, the increased level of testosterone and the activity of inflammatory cytokines were observed (24). Oreillyet al reported that high levels of androgens were sensitive indices to predict MS in women with PCOS (13). Also it was reported that hyperandrogenism in PCOS patients with metabolic disorders played an important role. The prevalence of MS, insulin resistance and serum lipids were significantly higher in patients with hyperandrogenism compared to patients without hyperandrogenism (25). Mandrella et al also noted higher free testosterone levels in PCOS women with MS compared to normal ones (19). It seems that these differences might be due to differences in genetics, lifestyle and socioeconomic conditions, and sample size.

Also Munzker et al mentioned higher levels of total and free testosterone and DHT in patients with PCOS compared with the control group. They also observed that the TT / DHT in patients with PCOS were significantly higher in women with obesity, MS, glucose intolerance, and insulin resistance. In this study, TT / DHT has been noted as a good marker to assess severe metabolic outcomes in patients with PCOS (26).

Yet there is uncertainty about the normal androgens limit, the selected androgens, and techniques used to analyze it (22). The most accurate method to determine the level of androgens in PCOS patients is collecting 24-hours urine for assessing metabolites of androgens (27), but this method of measurement is very time consuming and costly. Therefore, commonly total testosterone is assessed as an accessible marker for detecting increased chemical androgen (2) and these differences might be as a result of uncertainty in diagnosing more useful androgens and different methods of measurement.

 In the relationship between testosterone with components of MS, results showed that the mean free testosterone levels was significantly related with diastolic blood pressure, high blood pressure and cholesterol levels in people with MS. In patients with diastolic blood pressure ≥ 85 and also with blood pressure ≥ 130/85, significantly higher testosterone levels were noted and with increasing levels of testosterone, cholesterol levels increased. 

However, inconsistent result was noted by Huisman et al (28). They noted higher levels of testosterone in hypertensive males and females comparing normotensives. Lower level of TG and higher level of LDL were also noted in males with high testosterone level. Moreover, in women with higher level of testosterone, increased cortisol level and renin system were noted. Due to higher activity of the renin system in women, it seems that high levels of testosterone might have a role in the renin-angiotensin system and testosterone increased blood pressure through the renin angiotensin system (28).

Evidence from animal and clinical studies showed that testosterone had beneficial effects on vascular reactions, inflammation, lipid concentration, and hemostatic factors and can affect cardiovascular health. According to the prevalence of MS in women with PCOS and its association with atherosclerotic diseases, it is predicted that it might induce 7 times increased risk of cardiovascular diseases in this group of women (28).

In the present study, there was a significant correlation between free testosterone level and cholesterol, so that by increasing testosterone levels, increased cholesterol levels were noted and vice versa. Velasco et al also mentioned high prevalence of hypercholesterolemia (26%) (29). Since, women with PCOS are more likely to have MS, specific strategies are needed to prevent the development of the disease and its complications in women with PCOS. Weight loss interventions such as diet, exercise, and insulin sensitizers and anti-androgens may decrease the risk of cardiovascular disease and progressive diabetes in patients with PCOS.

Regarding the relationship between blood pressure and high levels of free testosterone, it seems that regular blood pressure screening has a higher priority of concern comparing other complications for preventing cardiovascular adverse effects in women with PCOS and hyperandrogenism.

## Conclusion

In our study by increasing in testosterone level, cholesterol and blood pressure were increased in women with PCOS and MS. It can be confirmed that high levels of androgens are sensitive indices to predict MS in women with PCOS.
